# Os acromiale may be a contraindication of the clavicle hook plate: case reports and literature review

**DOI:** 10.1186/s12891-021-04841-1

**Published:** 2021-11-22

**Authors:** Qi Sun, Ming Cai, Xiaoming Wu

**Affiliations:** 1 Department of Orthopaedics, Shanghai Tenth People’s Hospital, School of Medicine, Tongji University, 200072 Shanghai, China; 2 Department of Orthopedics, Shanghai First People’s Hospital, Shanghai Jiao Tong University, 200080 Shanghai, China

**Keywords:** Os acromiale, Clavicle hook plate, Distal clavicle fractures, Acromioclavicular joint dislocation, Case report

## Abstract

**Background:**

Os acromiale can be potentially missed or misdiagnosed as acromion fracture, and this can affect treatment determination if it is complicated with an ipsilateral shoulder injury. The clavicle hook plate is a widely used technique for distal clavicle injuries, leading to transacromial erosion, particularly when in the presence of os acromiale.

**Case presentation:**

A 70-year-old man and a 78-year-old man who had limited mobility and severe pain in their right shoulders following falls attended the emergency center. Both patients were diagnosed with os acromiale with CT or MRI and acute distal clavicle fracture or acromioclavicular joint dislocation. Following a comprehensive evaluation, os acromiale may limit the application of a clavicle hook plate due to potential transacromial erosion. The distal clavicle fracture with ipsilateral os acromiale received treatment with a volar radius locking T plate, and the acromioclavicular joint dislocation with ipsilateral os acromiale was reconstructed using suture anchors. Both yielded satisfactory outcomes and voided transacromial erosion.

**Conclusions:**

Ipsilateral os acromiale may be a relative contraindication to the clavicle hook plate. An axillary lateral radiograph is recommended to detect potential os acromiale in patients using a hook plate.

## Background

Os acromiale represents one or more unfused ossification centers of the acromion, and its frequency is documented in a range of 1–15 % [1–4]. Despite the reported symptom of shoulder pain, most of os acromiale is asymptomatic and discovered accidentally [4, 5]. Os acromiale can be potentially missed or misdiagnosed as acromion fracture, so therefore, two correct and clear views (anteroposterior and axillary views) are required to facilitate correct diagnosis [4]. Regarding symptomatic os acromiale, conservative treatments, such as physical therapy and subacromial corticosteroid injections, are commonly recommended [6]. Surgical interventions, including open fragment excision or arthroscopic decompression, can be used if conservative treatments fail [6]. Specifically, surgical intervention is required if os acromiale is complicated with ipsilateral shoulder injuries, such as distal clavicle fractures or acromioclavicular (AC) joint dislocation [7]. Previous literature has reported that transacromial erosion was caused by a subacromial hook plate in the treatment of AC joint dislocation, requiring the removal of the hook plate and suture reconstruction of the AC joint [8]. Therefore, os acromiale can affect treatment determination of distal clavicle fractures or AC joint dislocation, leading to different outcomes and complications.

**Fig. 1:**
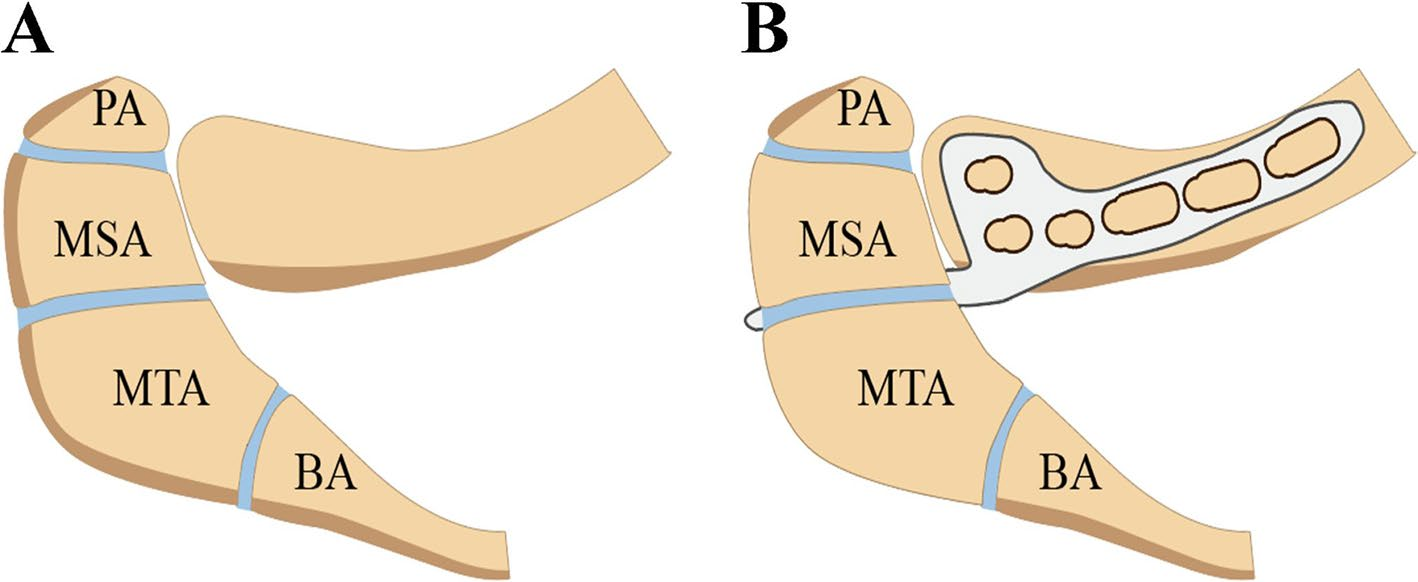
Schematic illustration of the positional relation between os acromiale and the clavicle hook plate. **A** The types of os acromiale. os PA: the space between the PA and MSA; os MSA: the space between the MSA and MTA; os MTA: the space between the MTA and BA. **B** The relative position of the clavicle hook plate to os acromiale. Pre-acromion: PA; Meso-acromion: MSA; Meta-acromion: MTA; Baso-acromion: BA. (The schematic image is drawn by the authors using Adobe Illustrator Version CC 2017).

The clavicle hook plate is a widely used technique for both distal clavicle fractures and AC joint dislocation, and it yields satisfactory clinical outcomes, despite lacking a gold standard [7, 9, 10]. Previous literature has demonstrated that the most common type of os acromiale is the unfused center between the meso-acromion (MSA) and meta-acromion (MTA) (Fig. 1A), where the coronal cleft is right at the posterior margin of the AC joint [11] and above the hook of the clavicle hook plate. Therefore, a clavicle hook plate can lead to transacromial erosion in the treatment of distal clavicle fractures or AC joint dislocation with os acromiale (Fig. 1B), despite os acromiale not listed or mentioned as a contraindication of a clavicle hook plate. Here, we present a unique case of os acromiale with distal clavicle fractures and a case of os acromiale with AC joint dislocation; both yielded satisfactory outcomes following surgical treatment.

## Case presentation

### Case 1

**Fig. 2:**
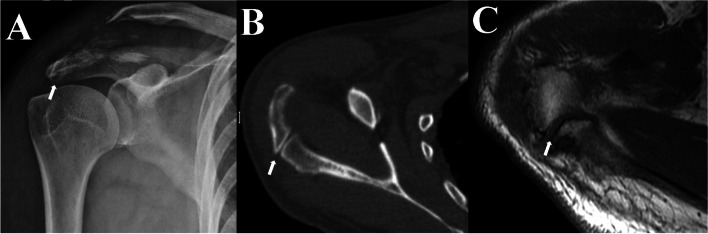
Preoperative examination of the right shoulder. **A** Right shoulder AP view: The displaced distal clavicle fracture with increased coracoclavicular distance. The os acromiale cannot be observed. **B** CT of the right shoulder: the clear sclerotic margin between the os acromiale and the truncated acromion. **C** Pre-operation MRI: it is likely that the interface between the os acromiale and the truncated acromion is a type of fibrocartilage. The white arrowhead indicates the os acromiale

A 70-year-old, right-hand dominant man attended the emergency center with limited mobility and severe pain in his right shoulder following a fall. The true AP plain radiograph revealed a displaced fracture of the right distal clavicle, a double-density sign with the cortical margin of MSA, and increased coracoclavicular distance (Fig. 2A). Upon admission, a computed tomography (CT) scan of the right shoulder was performed for a comprehensive evaluation, revealing a displaced, comminuted fracture to the distal right clavicle (Fig. 2B). The CT also showed incomplete fusion of acromion processes at the MSA site(Fig. 2B). For confirming whether the incomplete fusion was os acromiale, magnetic resonance imaging (MRI) was performed, indicating that the interface between the native acromion and unfused ossification center was most likely presented as a type of fibrocartilage tissue (Fig. 2C).

**Fig. 3:**
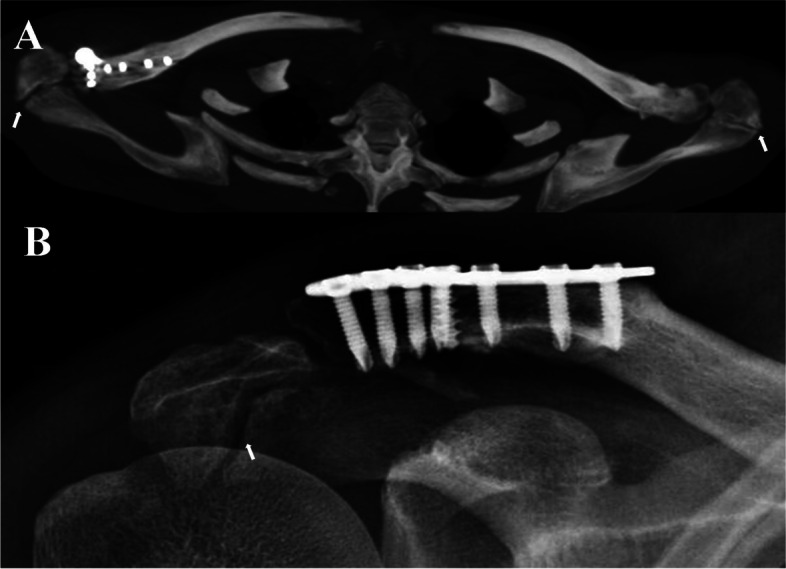
Postoperative CT and X-ray. **A** Bilateral shoulder CT: the distal clavicle fracture is reduced and fixed using a 3.5 mm distal radius volar plate. The os acromiale is bilateral in this case. **B** Two years after surgery, the true AP view: the distal clavicle fracture achieved bone union in the anatomic position. The os acromiale can be observed. The white arrowhead indicates the os acromiale

On day three after the injury, the patient received open reduction and internal fixation (ORIF) under general anesthesia. Following the dissection, the distal clavicle fracture was reduced under direct vision and subsequently fixed using a 3.5 mm T-shape distal radius volar locking plate (Acumed Acu-Loc® Wrist Plating System, Acumed Headquarters, Hillsboro, Oregon), which was adjusted to fit the contour of the distal clavicle with convergent screw direction (Fig. 3). The tear of the coracoclavicular ligament was repaired using an absorbable suture. Once the fixation stability and range of movement were confirmed, the wound was closed.

The patient started a pendulum movement of the shoulder with sling immobilization and active movement of the elbow and wrist one day after surgery. Active non-weight-bearing overhead movement of the right shoulder was initiated eight weeks after surgery. Normal shoulder movement was encouraged until radiological confirmation of bony union at week 12 postoperatively. During two years of follow-up, the patient had no particular complaints. At the last follow-up, the distal clavicle fracture achieved bony union. However, os acromiale remained visible (Fig. 3B). The patient achieved satisfactory shoulder function and muscle strength with a constant score of 95.

### Case 2

**Fig. 4:**
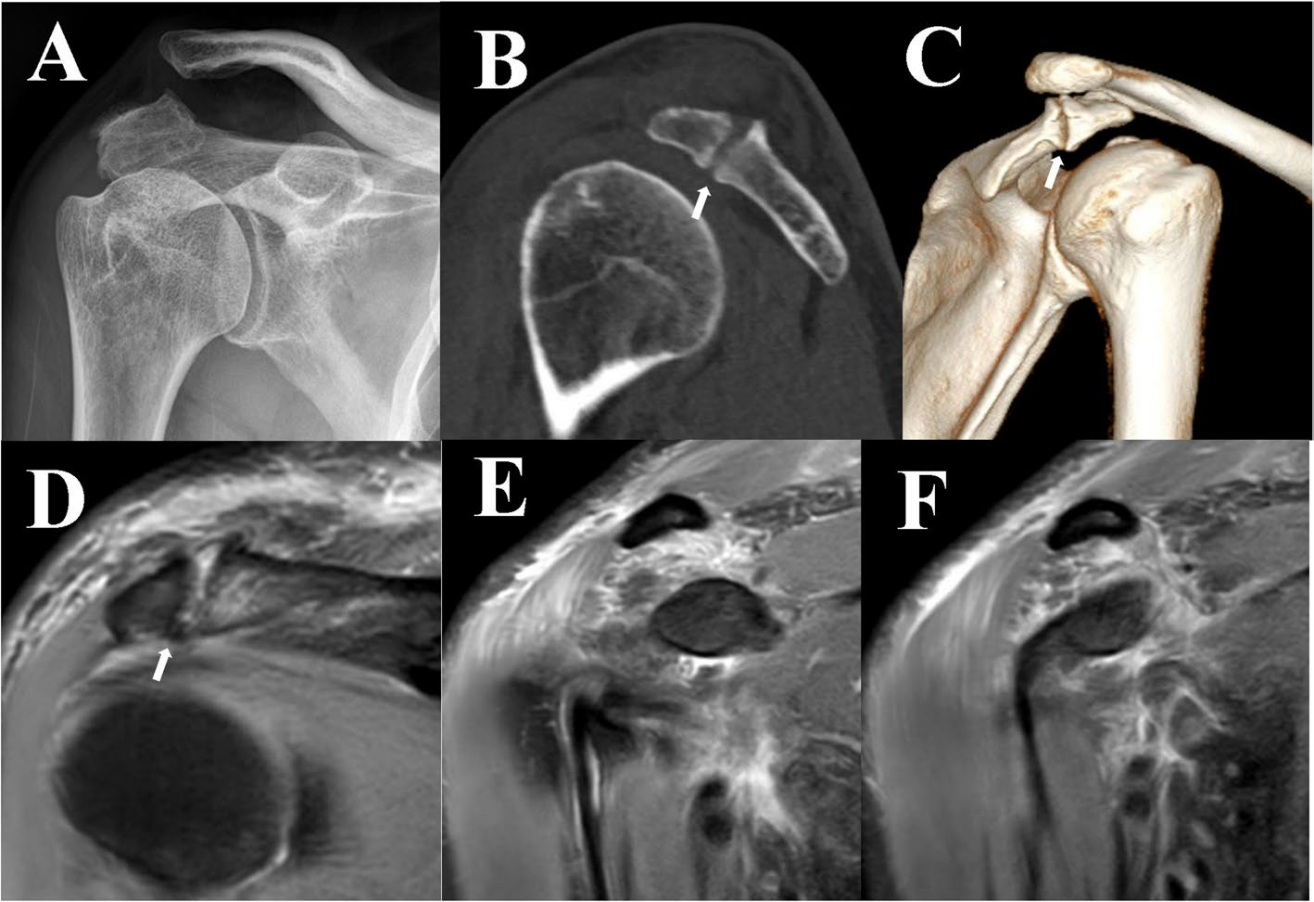
Preoperative examination of the right shoulder. **A** Right shoulder AP view: The increase of AC distance. **B**, **C** CT of the right shoulder: clear sclerotic margin between the os acromiale and the truncated acromion. **D** Pre-operation MRI: the interface between the os acromiale and the truncated acromion is likely a type of fibrocartilage. **E** Pre-operation MRI: discontinuity of the trapezoid ligament. **F** Pre-operation MRI: swollen and increased signal of conoid ligament. The white arrowhead indicates the os acromiale

A 78-year-old, right-hand dominant man suffered a fall, which resulted in limited mobility, severe pain, and deformity to his right shoulder. The true AP plain radiograph revealed obvious increased coracoclavicular distance and AC distance and a double-density sign with the cortical margin of MSA (Fig. 4A). We performed a CT scan with three-dimensional reconstruction following admission, which implied nonunion of the right acromion between the MSA and MTA (Fig. 4B and C). In order to confirm os acromiale and potential injury to the rotator cuff and adjacent ligaments, an MRI was performed on the patient’s right shoulder, indicating that the connection between the native acromion and unfused ossification center was a type of fibrocartilaginous tissue (Fig. 4D). In addition, the MRI demonstrated the discontinuity of the trapezoid ligament and increased signal of the conoid ligament (Fig. 4E and F).

On day four following the injury, the patient received ORIF under general anesthesia. Following dissection, debridement of the AC joint, and removal of the cartilaginous disc under direct vision, a 3.5 mm suture anchor with double-loaded sutures was inserted into the base of the coracoid process. The suture strands were passed through a hole in the clavicle created using a 2.0 mm drill and tied around its anterior border. The injured supraspinatus was repaired using one lateral and two medial row anchors (Arthrex, Naples, FL). The shoulder capsule was then released.

The rehabilitation strategy was similar to that used in case 1 until eight weeks postoperatively. Loss of reduction and complications were not observed during follow-up. The patient yielded satisfactory shoulder function with a constant score of 94 three months after surgery.

Both patients provided written informed consent. All work described above was carried out according to The Code of Ethics of the World Medical Association (Declaration of Helsinki), which applies to experiments involving human subjects (http://www.wma.net/en/30publications/10policies/b3/index.html).

## Discussion and conclusions

Although os acromiale is not commonly encountered in daily practice, the possibility exists that the patient with asymptomatic os acromiale suffered from AC joint dislocation or distal clavicle fracture [12]. As far as we know, the case report first described that asymptomatic os acromiale was diagnosed with an ipsilateral distal clavicle fracture, and the patient received locking plate fixation and achieved satisfactory shoulder function without any major complications. The AC joint dislocation with ipsilateral os acromiale was repaired using a suture anchor and augmented by non-absorbable heavy sutures, and this yielded an excellent outcome at the last follow-up appointment.

Synchondrosis between the os acromial and the truncated acromion varied from the fibrocartilaginous [13] to almost complete union [2], partly explaining its different clinical presentations. A conventional AP view of the shoulder cannot show the acromion well, and axillary radiographs played a critical role in the diagnosis of os acromiale. Os acromiale is sometimes missed, particularly when associated with a severe ipsilateral shoulder injury, and the radiography taken in the orthopedic emergency department was insufficient. In case 1, the AP view failed to detect the os acromiale when the patient attended the emergency center. The displaced distal clavicle fracture and non-compliance due to severe pain caused a misdiagnosis of os acromiale.

Os acromiale generally needs to be confirmed if there is a double-density sign in the AP view of cortical irregularity on the supraspinatus outlet view [14]. Notably, an axillary lateral radiograph of the shoulder is a valuable tool for detecting os acromiale [15]. In addition, if os acromiale is suspected, a CT scan will contribute to its detection and confirmation. Specifically, MRI is the most effective method for confirming os acromiale, and it helps define the character of the interface and differential diagnosis. In both cases, CT revealed a clear sclerotic margin between the os acromiale and the truncated acromion. MRI indicated that the interface between the os acromiale and the truncated acromion was a type of fibrocartilage, helping greatly in diagnosis confirmation. Another tip for avoiding missed diagnosis is understanding the anatomical variation. The characteristic of the higher incidence of 62 % with os acromiale bilaterally is helpful for differential diagnosis. The patient also exhibited bilateral os acromiale in case 1 (Fig. 3A).

Surgical intervention is recommended in cases with displaced distal clavicle fractures and AC joint dislocation because of a high rate of complications with conservative treatment [16]. Although the consensus on surgical treatments of distal clavicle fracture and AC joint dislocation remains controversial, surgical management is highly recommended. If a surgical strategy is determined and performed on ipsilateral shoulder injuries, neglect or missed diagnosis of os acromiale may lead to significant adverse events. Hook plate fixation is a widely used technique involving Neer type II lateral clavicle fractures and Tossy III AC joint dislocation [17–19]. A case report showed transacromial erosion was caused by a clavicle hook plate when treating AC joint dislocation [20]. An anatomic study demonstrated that 60 % of hook plates had focal contact with the undersurface of the acromion at the hook tip [21]. According to morphometric speculation, the hook tip should fit underneath and have focal contact with the nonunion interface of the os acromiale, particularly right below the unfused center between the MSA and MTA, potentially leading to transacromial erosion (or hook cut through the acromion) (Fig. 1A and B). In addition, a hook plate may require the removal of the implant to avoid breakage or bony erosion of acromion or because this has already occurred. Therefore, we believe that the hook plate should be excluded from the treatment of distal clavicle fractures or AC joint dislocation when complicated with ipsilateral os acromiale, particularly for the type of os MSA.

Distal clavicle fractures are characterized by small fragments and comminution, requiring stable fixation. The small size of the distal fragment limits the application of strong fixation constructs because of strong displacing forces on the proximal fragment induced by the counter-pull of the trapezius on its long moment arm [22]. Therefore, current fixation methods, including K-wire and lateral clavicle plate, can be problematic when treating distal clavicle fractures [22]. To achieve stable fixation and avoid any potential complications, a pre-contoured radius volar locking plate can be used to treat distal clavicle fracture with ipsilateral os acromiale, reported by the previous literature [23]. The oblique T-shaped locking plate with a low profile allows multiple locking screws into both the small distal fragment and the long proximal fragment and can conform to the anatomical shape of the clavicle, providing superior rigid fixation to K-wire and tension-band wires [23, 24].

Arthrex dog bone and tightrope fixation system are alternatives, but coracoclavicular fixation is not recommended as the first-line treatment of unstable distal clavicle fractures [25]. There is a high risk of iatrogenic fractures in anatomic coracoclavicular ligament reconstructions, and clavicular and coracoid bone tunnels cause a fracture risk of over 18 % in both locations. The smaller size of the coracoid process in Asians may potentially increase the incidence of iatrogenic coracoid fractures. To summarize, a novel anatomical locking plate for the distal clavicle is essential and is perhaps an essential direction for treating distal clavicle fractures in the future [23, 26].

Regarding the case with AC joint dislocation, a double row suture anchor and non-absorbable heavy sutures were used for repair and augmentation. The patient achieved satisfactory shoulder function during the follow-up and did not complain of pain or other complications. The reconstruction of AC joint dislocation using suture anchors has become an attractive alternative with the advantages of lower morbidity rates, exemption of hardware removal, and minimal complications of the breakage or migration of metal implants [27]. The technique yields reliable outcomes when treating AC joint dislocation, avoiding the potential damage of os acromiale.

We believe this to be the first description of the unmentioned contradiction of the clavicle hook plate due to the presence of os acromiale (Fig. 1). The ipsilateral distal clavicle fracture and AC joint dislocation achieved satisfactory shoulder function following treatment with a volar radius locking plate and suture anchors, respectively. The major limitation may be attributed to the fact that clavicle hook plate-oriented transacromial erosion was not reported in either case, resulting from careful evaluation and correct treatment determination. That is why we performed a literature review of the prevalence and morphology of os acromiale and proposed that the clavicle hook plate can potentially lead to transacromial erosion in the presence of os acromiale.

These cases highlight the need for appropriate radiographic investigation, including an axillary view and CT scan preoperatively and a comprehensive evaluation of the fixation. Although not mentioned in previous literature, caution should be exercised as ipsilateral os acromiale may be a relative contraindication to the clavicle hook plate when complicated with distal clavicle fractures or AC joint dislocation. Application of a clavicle hook plate in these circumstances will fail.

## Data Availability

The datasets used and analyzed during the current study are available from the corresponding author on reasonable request.

## Abbreviations

AC: Acromioclavicular
CT: Computed tomography
MRI: Magnetic Resonance Imaging
PA: Pre-acromion
MSA: Meso-acromion
MTA: Meta-acromion
BA: Baso-acromion
